# CD44 is a potential immunotherapeutic target and affects macrophage infiltration leading to poor prognosis

**DOI:** 10.1038/s41598-023-33915-4

**Published:** 2023-06-14

**Authors:** Shuangqing Liu, Zehan Liu, Aichen Shang, Jing Xun, Zongjing Lv, Siying Zhou, Cui Liu, Qi Zhang, Yuming Yang

**Affiliations:** 1 Tianjin Medical University Nankai Hospital, Tianjin, 300070 China; 2 Section for HepatoPancreatoBiliary Surgery, Department of General Surgery, The Third People’s Hospital of Chengdu, Affiliated Hospital of Southwest Jiaotong University and The Second Affiliated Hospital of Chengdu, Chongqing Medical University, Chengdu, 610031 China; 3 Department of Pathology, Sino-Singapore Eco-City Hospital of Tianjin Medical University, Tianjin, 300456 China; 4 Tianjin Key Laboratory of Acute Abdomen Disease Associated Organ Injury and ITCWM Repair, Institute of Acute Abdominal Diseases, Tianjin Nankai Hospital, Tianjin, China; 5 Department of Pathology, Tianjin Nankai Hospital, Tianjin, China

**Keywords:** Cancer therapy, Tumour immunology, Cancer, Immunology, Tumour immunology

## Abstract

CD44 plays a key role in the communication of CSCs with the microenvironment and the regulation of stem cell properties. UALCAN was used to analyze the expression of CD44 in bladder cancer (BLCA) and normal tissue. The UALCAN was utilized to analyze the prognostic value of CD44 in BLCA. The TIMER database was used to explore the relationship between CD44 and PD-L1; CD44 and tumor-infiltrating immune cells. The regulatory effect of CD44 on PD-L1 was verified by cell experiments in vitro. IHC confirmed the results of the bioinformatics analysis. GeneMania and Metascape were used to analyze protein–protein interaction (PPI) investigations and functional enrichment analysis. We found that BLCA patients with high CD44 expression had worse survival than those with low CD44 expression (*P* < 0.05). IHC and the TIMER database results showed that CD44 expression was positively correlated with PD-L1 expression (*P* < 0.05). At the cellular level, the expression of PD-L1 was significantly inhibited after CD44 expression was inhibited by siRNA. Immune infiltration analysis showed that CD44 expression levels in BLCA were significantly correlated with immune infiltration levels of different immune cells. IHC staining results further confirmed that the expression of CD44 in tumor cells was positively associated with the number of CD68^+^ macrophages and CD163^+^ macrophages (*P* < 0.05). Our results suggest that CD44 is a positive regulator of PD-L1 in BLCA and may be a key regulator of tumor macrophages infiltration and may be involved in M2 macrophage polarization. Our study provided new insights into the prognosis and immunotherapy of BLCA patients through macrophage infiltration and immune checkpoints.

## Introduction

According to the European Association of Urology (EAU) guidelines, bladder cancer is divided into non-muscle-invasive bladder cancer (NMIBC) and muscle-invasive bladder cancer (MIBC). BLCA can also be divided into urothelial carcinoma, squamous cell carcinoma, and adenocarcinoma, of which more than 90% are urothelial carcinoma^1^. Among them, NMIBC patients account for nearly 80% of newly diagnosed cases and are prone to the risk of recurrence (~ 70%) and progression (~ 15%) after standard treatment according to clinical guidelines^2^. BLCA is the twelfth most common cancer worldwide, with 549,393 new cases (representing 3% of all new cancers) estimated in 2018, and the fourteenth most common cause of death from cancer with 199,922 deaths (representing 2.1% of all new cancers)^3^. The total cost of BLCA in the USA in 2010 was $US4 billion and is likely to grow to $US5 billion by 2020^4^. It can be seen that BLCA has brought a huge burden to patients and society, and it is of great significance for BLCA patients to seek better treatment strategies.

The CSCs theory states that tumor growth is almost driven by a small number of cancer stem cells dormant in the cancer^5^. CSCs signaling has been a focus of early-stage therapeutic development efforts for clinical evaluation^6^.

CD44 is a member of the hyaluronic acid (HA) receptor family of cell adhesion molecules. The primary ligand is hyaluronate, an abundant extracellular polysaccharide found in the extracellular matrix (ECM), but CD44 has many different functions, depending on the protein’s extracellular structure, and can produce multiple isoforms^7^. There is increasing evidence that some CD44 isoforms provide potential prognostic biomarkers and therapeutic targets for many cancers^8^. Recent studies have shown that CD44 is the most common surface marker of CSCs and plays a key role in the communication of CSCs with the microenvironment and the regulation of stem cell properties^9^. It was found that the effect of TAM (tumor-associated macrophages) on CSCs function was dependent on HA-CD44 interaction and on CD44 isoform expression^10^. Tumor-associated fibroblasts enhance tumor stem cell properties by osteopotin/secreted phosphoprotein 1-CD44 axis in pancreatic cancer^11^. But these studies only focused on the relationship between certain immune cells and CD44. Our study explored the relationship between CD44 and infiltration of immune cells.

The concept of blocking inhibitory signals, known as checkpoints, is a major shift in the field of cancer immunotherapy^12^. In antitumor immunotherapy, PD-L1, CTLA-4, and CD47 are all targets of immune checkpoints^13^. PD-1 expressed on tumor-infiltrating immune cells and PD-L1 expressed on antigen-presenting cells and tumor cells are interacting immune checkpoint proteins that negatively regulate anti-tumor adaptive immune responses^14,15^. CTLA-4 was first identified in 1991 as a second receptor for the T cell costimulatory ligand B7^16^. Since then, CTLA-4 has achieved clinical success as a target for antitumor therapy and as a soluble inhibitor of autoimmunity^17^. One key mechanism of tumor cell immune escape is the overexpression of the immunosuppressive signaling molecule CD47^18–20^. Overexpression of CD47 on the surface of tumor cells can help these cells escape surveillance and clearance by immune cells, making CD47 a potential target for the development of new anticancer drugs^21^.

In this study, CD44 was examined in the context of predictive value and immune functions using public databases. In our study, we sought to investigate the role of CD44 in the regulation of tumor cell immune evasion and immune cell infiltration, as well as provide novel clues for administering immunotherapy to BLCA patients.

## Materials and methods

### Databases and RNA-seq/gene expression analysis

The datasets for this study were obtained from public databases, including The Cancer Genome Atlas (TCGA) (https://cancergenome.nih.gov), the UALCAN database (https://ualcan.path.uab.edu)^22,23^, Tumor Immune Estimation Resource (TIMER) database (https://cistrome.org/TIMER/)^24,25^.

### RNA-seq and correlation analysis

The databases, TIMER, were used for mRNA/gene expression analysis in normal versus tumor tissues in several cancers. Furthermore, the UALCAN database was also used to study CD44 expressions correlation with patients’ prognosis in BLCA. Parameters were set as fold change required: > 1.5; gene rank: 10%.

### Tumor infiltration analysis

TIMER, a database of immune cells that infiltrate tumors, was used to investigate immune cell infiltration into tumors. The TIMER database uses TCGA data to calculate gene expression associations between cancer and immune infiltration. The correlation coefficients for gene expression were calculated with Spearman's correlation and displayed as log2 RSEM.

### Protein–protein interaction investigations and functional enrichment analysis

The GeneMANIA database (https://genemania.org)^26^ has been possible to construct protein–protein interaction (PPI) network and investigate function. GeneMANIA displays genes that are coexpressed, colocalized, and physically interact with CD44. We obtained the gene data related to BLCA on the GeneCards Suite Databases^27^. Feature enrichment analysis of genes that were in the intersection was performed using KEGG pathway analysis by Metascape (https://metascape.org)^28–30^.

### Patients and specimens

Our study included patients diagnosed with BLCA (n = 31). transurethral resection of bladder tumour. Immunohistochemical serial slides were obtained from formalin-fixed and paraffin-embedded sections of surgical tumor specimens according to standard clinical protocols. Tianjin Nankai Hospital granted Ethical approval to carry out the study within its facilities (Ethical Application Ref: NKYY_YXKT_IRB_2021_132_01). Due to the retrospective design of this study, the medical Ethics Committee of Tianjin Nankai Hospital approved the written informed consent requirement to be waived.

### Immunohistochemistry staining

All patients were diagnosed with bladder cancer. 26 BLCA patients underwent transurethral resection of the bladder tumor (TURBT) and 5 BLCA patients underwent radical cystectomy. The IHC protocol was performed by previous descriptions. Briefly, tumor sections were stained with rabbit anti-CD44 (abclonal, A19020-50), anti-CD68 (abclonal, A20803-50). The Goat anti-rabbit antibody (Bioss, SP-0022) was used as the secondary antibody. Scoring of the staining CD44 and CD68 immunostaining analysis was performed under a 400× microscope by two independent pathologists blinded to the patient’s clinical data. The staining sections of CD44 were then reviewed and scored as follows by a pathologist with over 15 years of experience: 10% of cells stained as positive staining (+, 1); 10–49% of cells stained as (+, 2); 50–74% of cells stained as (++, 3); stained 75–100% of cells were scored as (+++, 4). The dyeing color is divided into light yellow particles (1), brown-yellow particles (2), and brown particles (3)^31^. For quantification of CD68 positive cells and CD163 positive cells, 5 randomly selected regions (400× magnification) were counted and averaged to represent the number of positive cells in that fraction^32^.

### Cell culture and reagents

Human BLCA cell line 5637 was cultured in 1640 medium supplemented with 10% fetal bovine serum (Biological Industries, 04-001-1ACS) and 1% penicillin (Cytiva, SV30010) in a 5% CO_2_ incubator at 37 °C culture.

### siRNA transfection

CD44 siRNA oligonucleotides were purchased from Sheng Gong (Shanghai, China). siRNA transfection was performed using transfection reagent (Yeasen, 40806ES02) according to the manufacturer’s instructions. Knockdown efficiency was demonstrated by RT-PCR 2 days after transfection.

### Reverse transcription-PCR (RT-PCR)

Total RNA (2 µg) was reverse transcribed into cDNA using the Hifair^®^ First Strand cDNA Synthesis Kit (Yeasen, 11139ES60). The qPCR method was performed using Hieff^®^ qPCR SYBR Green Master Mix Reagent (Yeasen), and the reactions were performed under the conditions specified by the manufacturer. Relative mRNA expression was normalized by GAPDH as gene expression, and calculated by 2^−ΔΔCt^ method. The primers used include: GADPH, 5′-GGAGCGAGATCCCTCCAAAAT-3′(forward) and 5′-GGCTGTTGTCATACTTCTCATGG-3′ (reverse); CD44, 5′-CCCTGCTACCAGAGACCAAGAC-3′(forward) and 5′-GCAGGTTCCTTGTCTCATCAGC-3′ (reverse); PD-L1,5′‐CCAAGGCGCAGATCAAAGAGA‐3′ and 5′‐AGGACCCAGACTAGCAGCA‐3′.

### Protein–protein docking

Molecular docking is a bioinformatics tool used for in silico analysis for the prediction of binding modes of a ligand with a protein 3-D structure. Here, we used HDOCK to perform docking calculations to predict the preferred binding interactions between CD44 and the reported protein.

### Statistical analysis

Hazard ratios (HR) and p-values (log-rank test) are shown for all survival curves. Spearman’s correlation was utilized for the comparison between CD44 and all other genes, including data from TIMER. The correlation between CD44 and tumor-infiltrating immune cells was analyzed by partial correlation analysis. This method is useful when there are confounding variables that may affect the relationship. The TIMER database used partial correlation analysis to remove the effect of tumor purity on correlated variables^33^. Pearson’s correlation test was used to analyze the correlation between CD44 and other genes. Statistical significance was considered significant when *P* < 0.05 or as indicated with the data.

### Ethics approval and consent to participate

This study was approved by the Ethics Committee of Tianjin Integrative Medicine Hospital. This study was conducted in accordance to Declaration of Helsinki. Due to the retrospective design of this study, the medical Ethics Committee of Tianjin Nankai Hospital approved the written informed consent requirement to be waived.

## Results

### The role of CD44 in tumorigenesis and as a prognostic marker in BLCA

**Figure 1:**
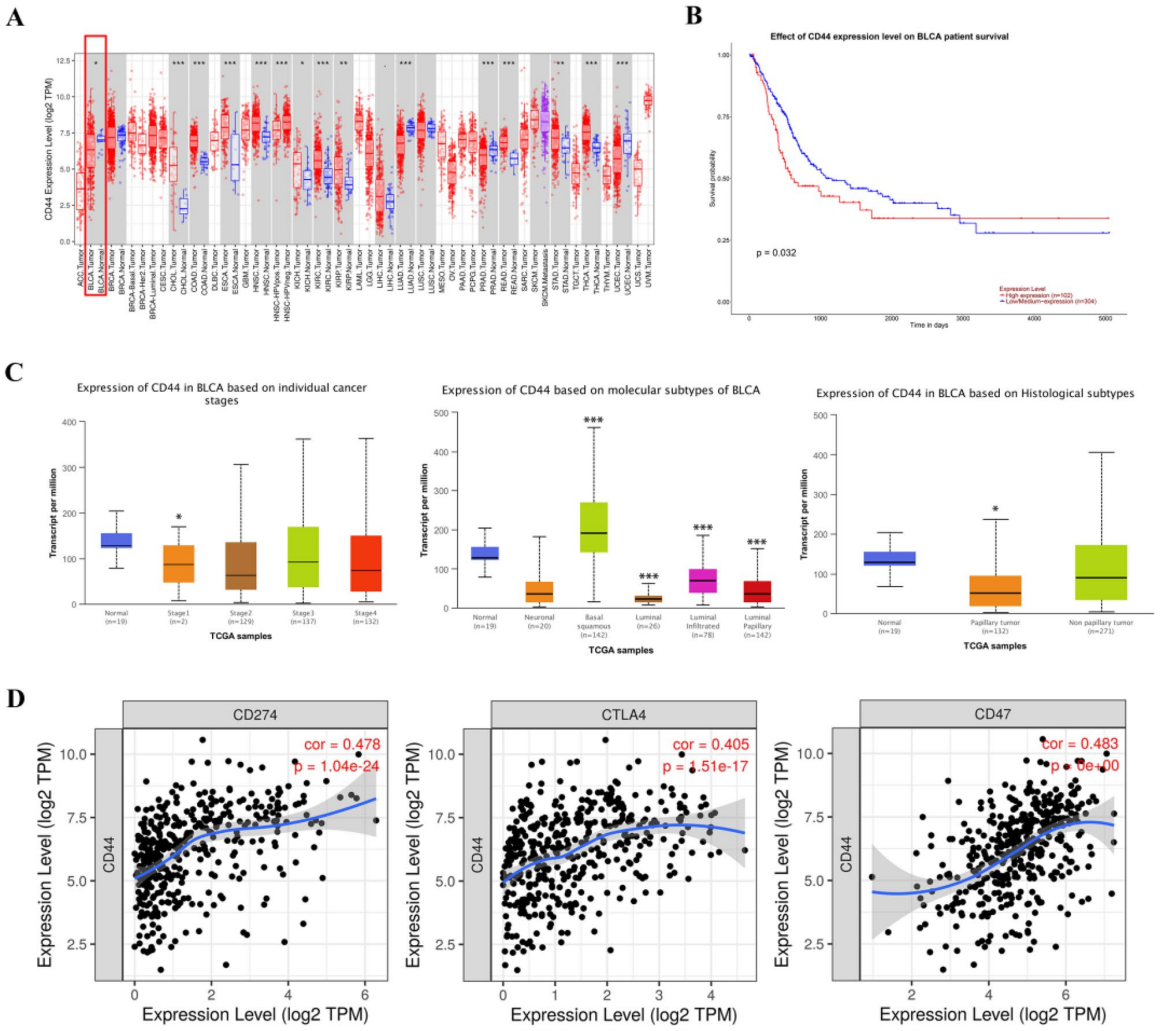
(**A**) Lower expression of CD44 was found in tumors as compared with non-tumor tissues in patients with BLCA. (**B**) Correlation between expression of CD44 and survival of 406 BLCA patients based on data from Ualcan plotter. (**C**) CD44 transcription in subgroups of patients with BLCA, stratified based on individual cancer stages, molecular subtypes, histological subtypes (UALCAN). (**D**) Correlation analyses between CD44 expression and Immune checkpoint-linked genes (PD-L1, CTLA-4, CD44) in BLCA using TIMER. (*P < 0.01, **P < 0.001, ***P < 0.0001).

In this study, we determined CD44 mRNA expression in normal and in tumor tissues for BLCA via the use of the TIMER database (Fig. 1A). Our analysis found that there was a significant difference in the expression of CD44 in BLCA and normal tissues. Kaplan–Meier method was used to analyze the survival rates of patients with high and low CD44 expression from the UALCAN database (Fig. 1B). As indicated by the survival curve, patients with high levels of CD44 mRNA expression had a worse prognosis than those with low levels of CD44 mRNA expression significantly (*P* = 0.032).

Further subgroup analysis of several clinicopathological features of TCGA BLCA specimens in the UALCAN database consistently showed differences in CD44 transcript levels (Fig. 1C). The expression level of tumor stage I was significantly lower than that of the normal group (*P* = 4.049900e−02). In all subtypes of BLCA, the expression of CD44 in basal squamous was significantly higher than that in normal subjects (*P* = 1.13710000304579e−08), the expression of CD44 in luminal type (*P* = 3.45430017922865e−10), luminal infiltrated (*P* = 3.185100e−04), luminal papillary (*P* = 1.42489999799267e−08) was significantly lower than that in normal subjects. In histology types of BLCA, the expression of CD44 in papilary tumor was significantly lower than that in normal subjects (*P* = 1.42489999799267E−08).

### CD44 expression is positively correlated with immune checkpoint marker in BLCA

We further investigated the association between CD44 expression and immune checkpoint marker (Fig. 1D). Through the use of the TIMER database, our analysis revealed a significant positive correlation between CD44 expression and PD-L1 (CD274: r = 0.478, *P* = 1.04e−24), CTLA4 (r = 0.405, *P* = 1.51e−24), CD47 (r = 0.483, *P* = 0e + 0). However, gene expression profiling of bulk tissues from TCGA fails to distinguish tumor cells from infiltrating immune cells. Therefore, we used the Human Protein Atlas website (https://www.proteinatlas.org/) to analyze the expression of CTLA-4 in immune cells and urothelial cells. CTLA-4 is mainly expressed in T cells, and the expression of CTLA-4 in urothelial cells is low (Fig. S1A,B). Therefore, we speculate that CD44 of tumor cells may affect the expression of CTLA-4 of T cells to help tumor cells immune evasion. These results suggest that CD44 expression may be related to the immune escape of tumor cells.

### CD44 is a novel positive regulator of PD-L1 in bladder cancer

**Figure 2:**
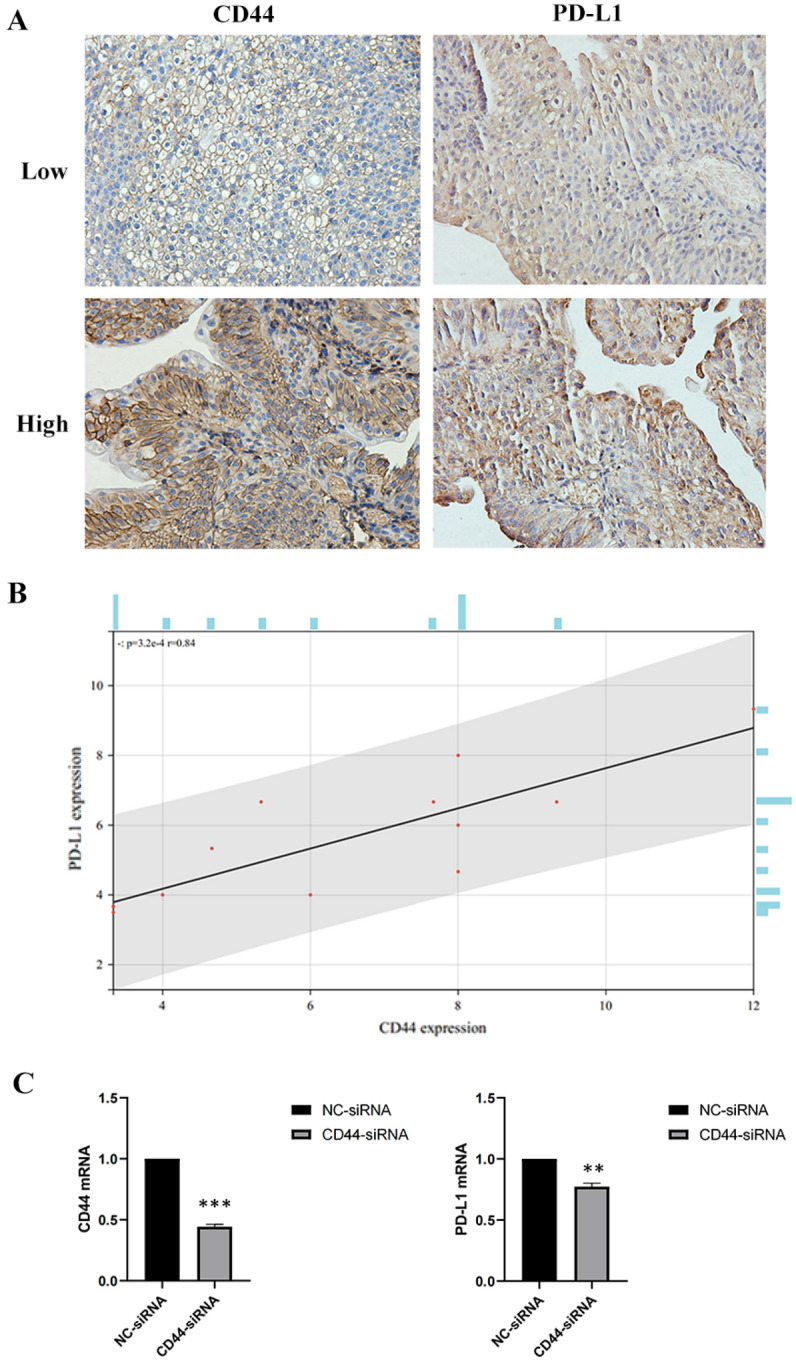
(**A**) representative IHC staining of CD44, PD-L1 in BLCA patient tumor sample. Upper: representative patient with high CD44 expression. Lower: representative patient with low CD44 expression. (**B**) Correlation analysis between the CD44 expression and the PD-L1 expression. Pearson’s correlation coefficient (r) and significance level (P-value) are shown as correlations. (**C**) 5637 cells were transfected with negative control (NC) siRNA or CD44 siRNA. mRNA levels in the transfected cells were assessed by RT-qPCR analysis.

To investigate whether there is a regulatory relationship between CD44 and PD-L1, we stained select BLCA samples by IHC for the CD44 and PD-L1 (Fig. 2A). We found that high CD44 expression of tumor cell expressed significantly higher levels of PD-L1 expression compared to low CD44 expression. Pearson correlation tests showed that CD44 expression was positively correlated with PD-L1 expression (r = 0.84, *P* = 3.2e−4) in BLCA (Fig. 2B). We knocked down CD44 using siRNA in the 5637 cell line, and CD44 suppression resulted in a decrease of PD-L1 mRNA expression (Fig. 2C).

### CD44 expression is associated with immune cell infiltration in BLCA and immune cell infiltration is related to the prognosis of patients

**Figure 3:**
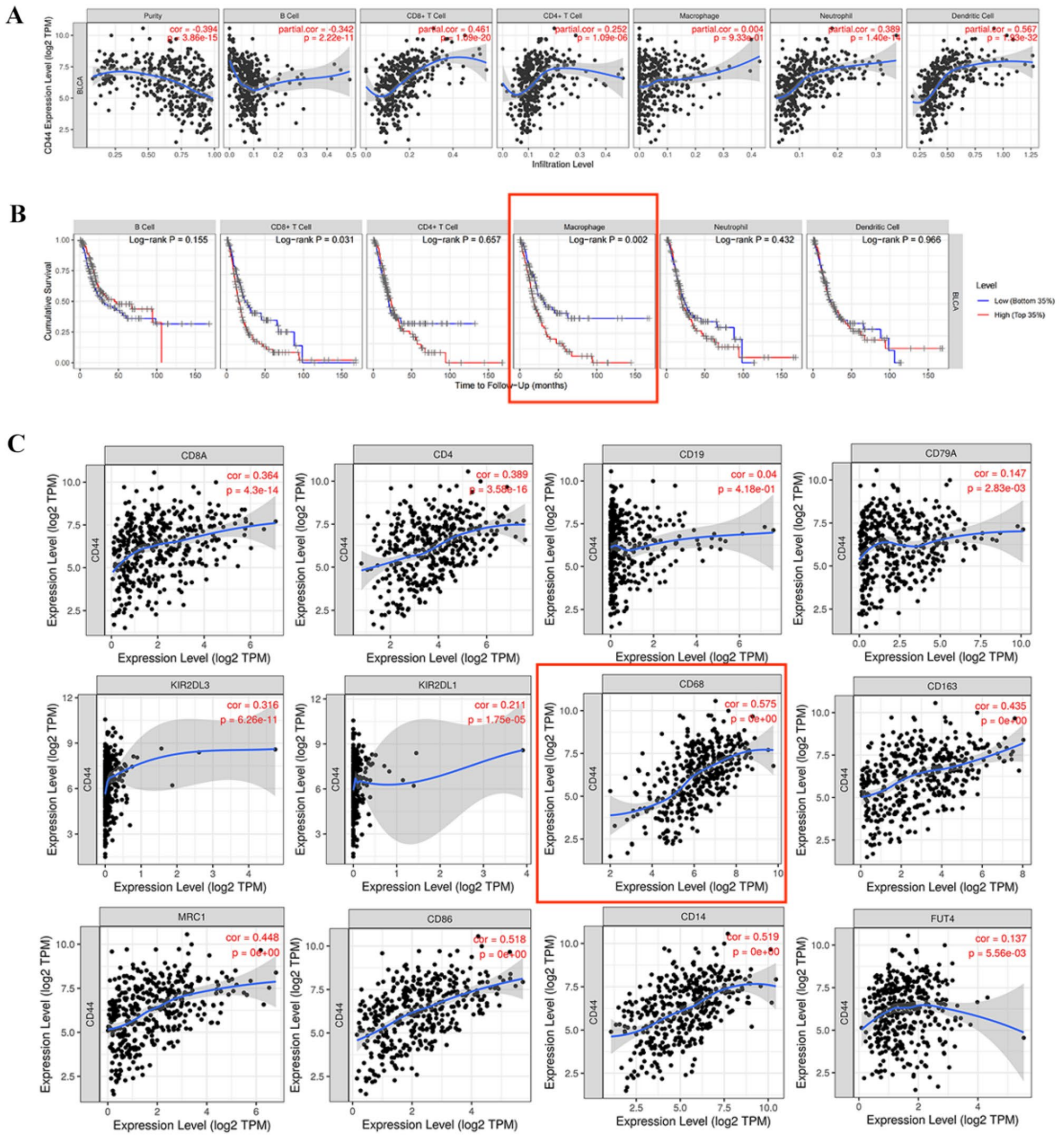
(**A**) CD44 expression is correlated with the level of immune infiltration in BLCA. (**B**) Kaplan–Meier plots of immune infiltration in BLCA. (**C**) Scatterplots of correlations between CD44 expression and gene markers of CD8 + T cell, CD4 + T cell, B cell, NK cell, macrophage, M1 and M2 macrophage, myeloid-derived suppressor cell exhaustion in BLCA.

In this study, TIMER was used to investigate the relationship between CD44 expression, and immune cell infiltration into the tumor in BLCA (Fig. 3A). Results have found that CD44 was positively correlated with CD8^+^ T cell (r = 0.461, *P* = 1.09e−20), CD4^+^ T cell (r = 0.252,* P* = 1.09e−06), neutrophil (r = 0.389, *P* = 1.40e−14) and dendritic cell (r = 0.567, *P* = 1.93e−32); CD44 was negatively with B cell (r = − 0.342, *P* = 2.22e−11). To examine the relationship between immune cells infiltration and prognosis in BLCA patients, we constructed Kaplan–Meier plots using the TIMER database (Fig. 3B). We found that macrophage infiltration (*P* = 0.002) was significantly associated with BLCA prognosis.

Next, we further examined the relationship between CD44 expression and BLCA immune cell marker using the TIMER database (Fig. 3C). We assessed the correlation of CD44 expression with specific cell subsets including CD8^+^ T cell (CD8A), CD4^+^ T cell (CD4), B cell (CD19, CD79a), NK cell (KIR2DL3, KIR2DL1), macrophage (CD68), M1 (CD86), and M2 (CD163, MRC1) macrophage, myeloid-derived suppressor cell (CD14, FUT4). The expression of CD44 in BLCA was positively correlated with the expression of markers for macrophage (r = 0.575, *P* = 0e + 00), M1 macrophage (r = 0.528, *P* = 0e + 00), M2 macrophage (CD163: r = 0.435, *P* = 0e + 00; MRC1: r = 0.448, *P* = 0e + 00), myeloid-derived suppressor cell (CD14: r = 0.519, *P* = 0e + 00). However, CD44 expression was not associated with macrophage infiltration in Fig. 3A. This is in contrast to the results in Fig. 3C. TIMER uses a published deconvolution method to infer the frequency of tumor-infiltrating immune cells from gene expression profiles^25^. Therefore, the results in Fig. 3A are estimated and may differ from the actual situation of tumor samples. This may explain the difference of results between Fig. 3A,C. And the relationship between CD44 expression and macrophage infiltration in bladder cancer needs experimental verification. In a word, these data suggest that CD44 expression is closely associated with immune infiltration in BLCA.

**Figure 4:**
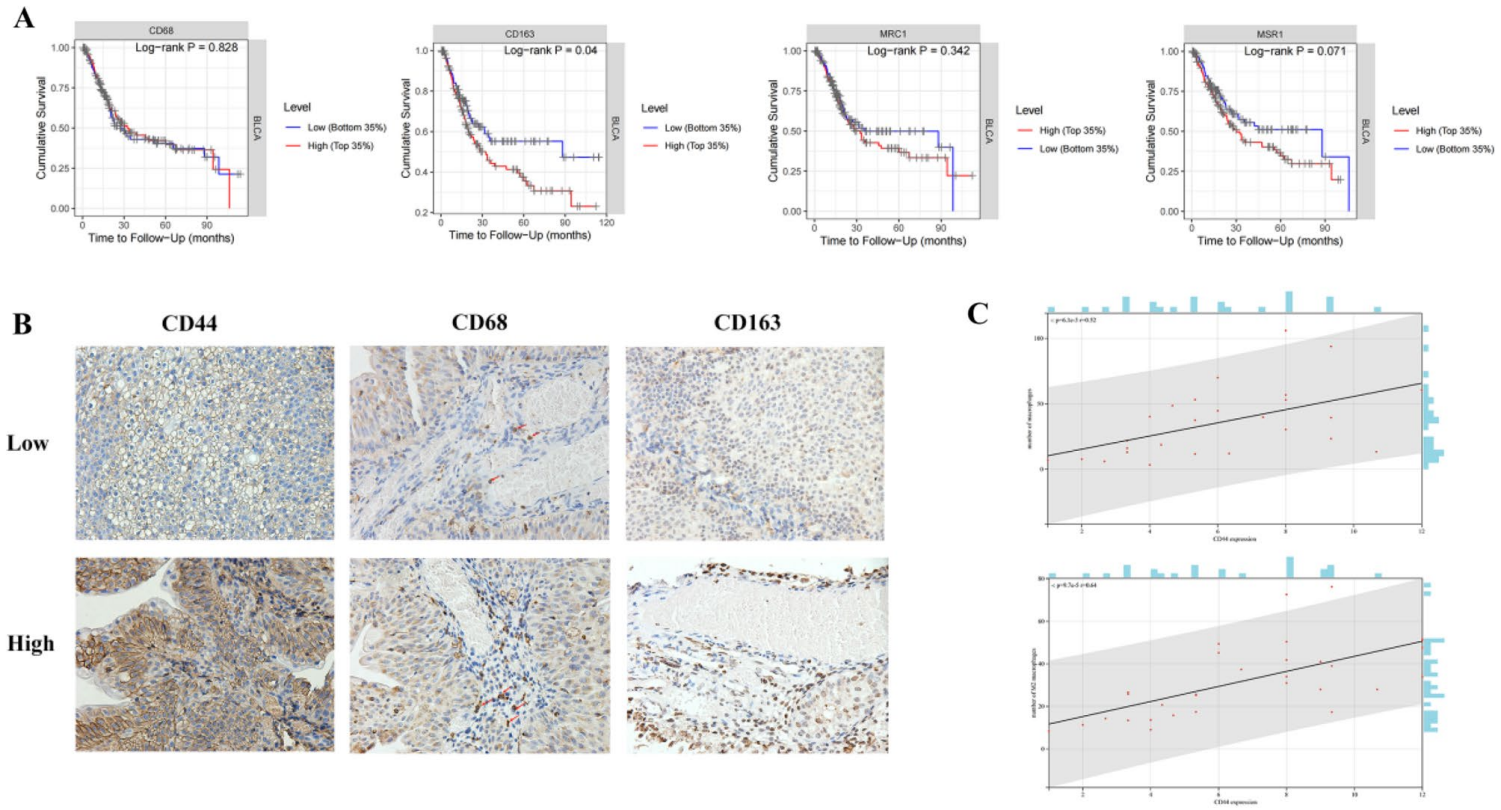
(**A**) Correlation between expression of gene markers of macrophage, M2 macrophage and survival of BLCA patients based on data from Kaplan–Meier plotter. (**B**) Representative IHC staining of CD44, CD68, and CD163 protein in BLCA tumor sample. Upper: representative patient with high CD44 expression. Lower: representative patient with low CD44 expression. (**C**) Correlation analysis between the percentage of CD44 and the number of macrophages and M2 macrophages per patient. Pearson’s correlation coefficient (r) and significance level (*P*-value) are shown as correlations.

We further analyzed the relationship between the expression of markers of macrophages (CD68), M2 macrophages (CD163, CD206, CD204 ) and the prognosis of BLCA using TIMER database (Fig. 4A). We found that CD68 expression was not associated with the prognosis, higher expression of CD163 (*P* = 0.04), CD206 (*P* = 0.342) and CD204 (*P* = 0.071) was significantly associated with worse prognosis in BLCA. To further verify the results of the database analysis, we stained select BLCA samples by IHC for the CD44 and TAM marker (CD68) and performed clinical pathological analysis (Fig. 4B–C). The results showed that samples with high CD44 expression had more CD68^+^ macrophage and CD163^+^ M2 macrophage infiltration than those with low CD44 expression (Fig. 4B). Pearson correlation tests showed that CD44 expression was positively correlated with the infiltration densities of CD68^+^ macrophages (r = 0.52, P < 0.01) and CD163^+^ macrophages (r = 0.64, *P* < 0.01) in BLCA (Fig. 4C). The clinical and pathological information of the patients are listed in Table 1.

**Table 1:** Clinical parameters of BLCA patients.

Variable	No. of patients (%)
Sex	
Men	23 (74.2)
Women	8 (25.8)
Age	
≤ 65	15 (48.4)
> 65	16 (51.6)
Tumor grade	
Poorly differentiated (high grade)	13 (41.9)
Well differentiated (low grade)	18 (58.1)
T stage	
Ta	11 (26.8)
T1	17 (41.4)
T2	3 (7.3)
T3–T4	0 (0.0)
Surgery type	
TURBT	26 (83.9)
Radical cystectomy	5 (16.1)

*TURBT* transurethral resection of bladder tumor.

### CD44 protein–protein interaction network and functional enrichment in BLCA

**Figure 5:**
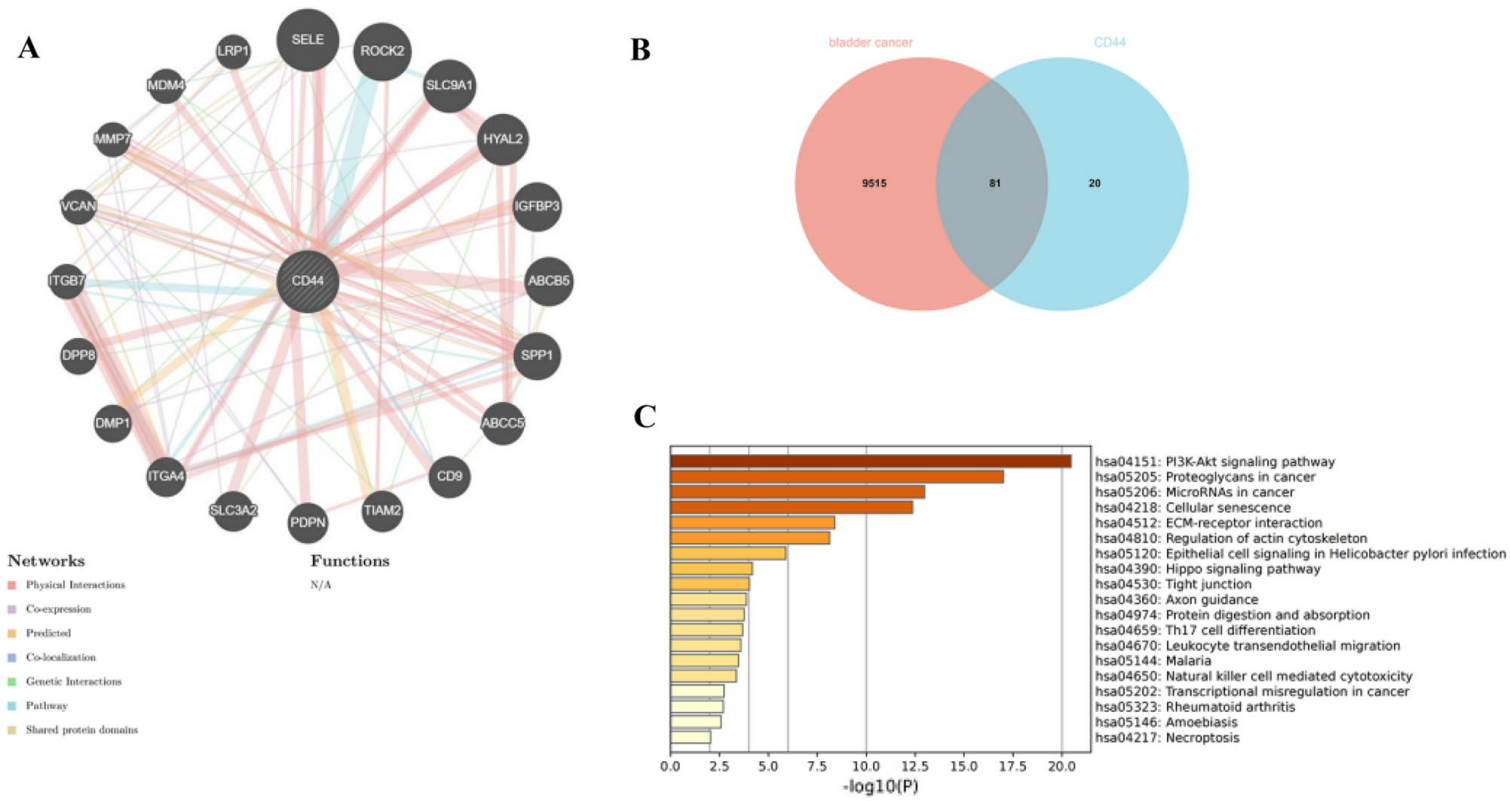
PPI networks and functional enrichment analysis of CD44; (**A**) PPI network of CD44 as depicted in GeneMANIA; (**B**) the intersection of Bladder cancer-related genes and genes that interact with CD44; (**C**) Feature enrichment analysis of the intersection of Bladder cancer-related genes and genes that interact with CD44.

It is important to identify CD44-interacting proteins and functional information about CD44-interacting genes. The protein–protein interaction (PPI) network of CD44 was analyzed using GeneMANIA (Fig. 5A). This online tool can analyze the genes related to CD44 from co-expression, physical interaction, genetic interaction, shared protein domains, pathway data, predicted function and other aspects. Different connection colors represent different ways of association with CD44 gene. Our analysis shows that CD44 interacts with SELE, ROCK2, SLC9A1, HYAL2, IGFBP3, ABCB5, SPP1, ABCC5, CD9, TIAM2, PDPN, SLC3A2, ITGA4, DMP1, DPP8, ITGB7, VCAN, MMP7, MDM4, and LRP1. Through searching bladder cancer-related genes in the GeneCards database, 9515 disease-related genes were obtained. The interaction analysis of the genes that interact with CD44 and bladder cancer-related genes was carried out, and 81 intersection target genes were obtained (Fig. 5B). In order to further explore the pathway mechanism of CD44 and bladder cancer, KEGG pathway analysis was carried out by Metascape. The three most significant enriched KEGG pathways are (i), PI3K-Akt signaling pathway (ii), Proteoglycans in cancer and (iii), MicroRNAs in cancer (Fig. 5C). It suggests that CD44 may affect bladder cancer through the above pathways.

### Protein–protein docking of CD44 and reported protein network

**Figure 6:**
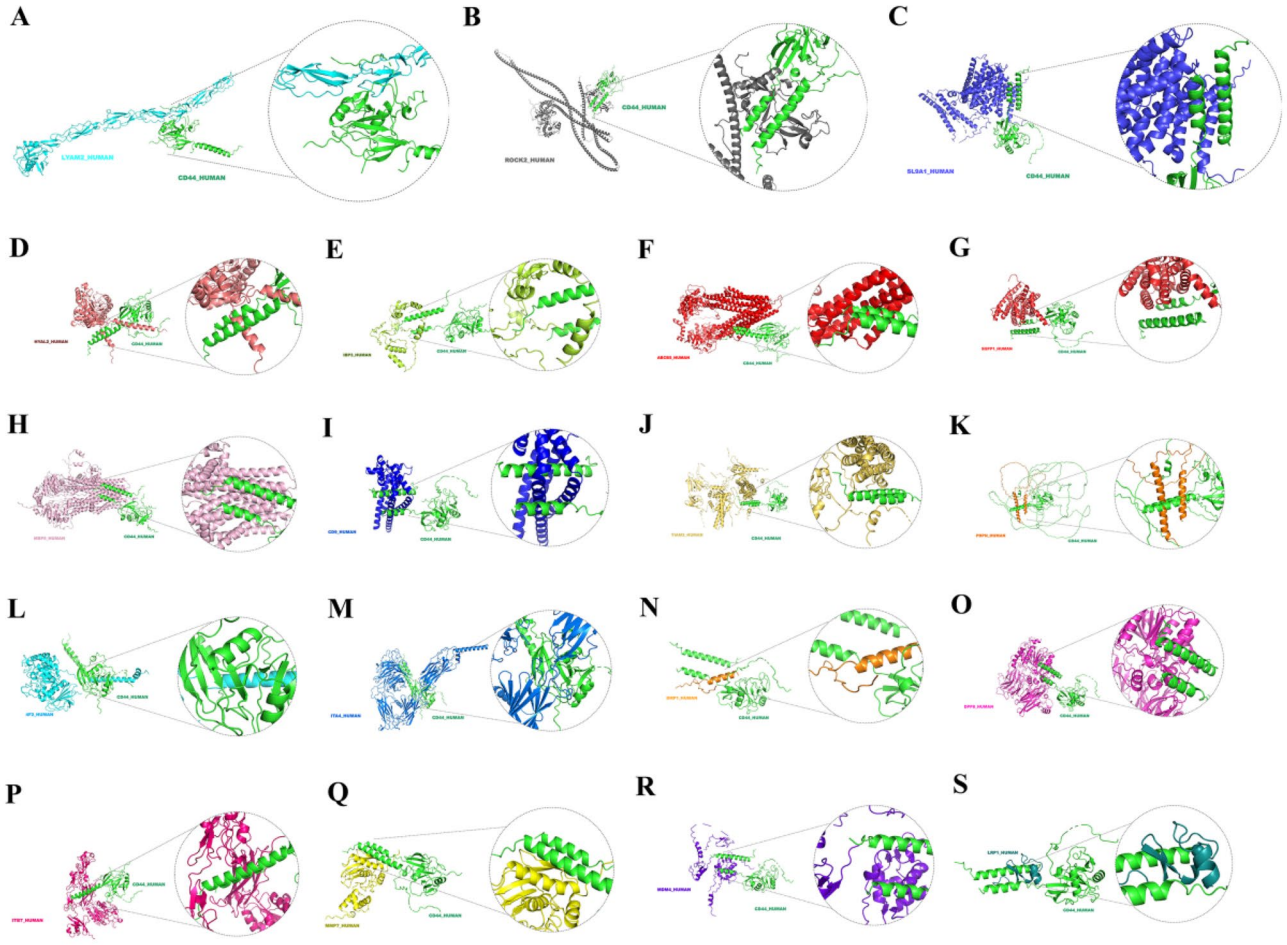
We performed docking calculations using HDOCK to predict preferred binding interactions between CD44 and 19 proteins reported in the PPI network. (**A**) Docking result of CD44 and LYAM (SELE) (docking score = − 243.65). (**B**) Docking result of CD44 and ROCK2 (docking score = − 243.06). (**C**) Docking result of CD44 and SL9A1 (SLC9A1) (docking score = − 357.36). (**D**) Docking result of CD44 and HYAL2 (docking score = − 316.46). (**E**) Docking result of CD44 and IBP3 (IGFBP3) (docking score = − 306.46). (**F**) Docking result of CD44 and ABCB5 (docking score = − 335.79). (**G**) Docking result of CD44 and SGPP1 (SPP1) (docking score = − 365.2). (**H**) Docking result of CD44 and MRP5 (ABCC5) (docking score = − 327.86). (**I**) Docking result of CD44 and CD9 (docking score = − 339.59). (**J**) Docking result of CD44 and TIAM2 (docking score = − 297.61). (**K**) Docking result of CD44 and PDPN (docking score = − 337.93). (**L**) Docking result of CD44 and 4F2 (SLC3A2) (docking score = − 273.52). (**M**) Docking result of CD44 and ITA4 (ITGA4) (docking score = − 283.97). (**N**) Docking result of CD44 and DMP1 (docking score = − 247.6). (**O**) Docking result of CD44 and DPP8 (docking score = − 294.82). (**P**) Docking result of CD44 and ITB7 (docking score = − 293.78). (**Q**) Docking result of CD44 and MMP7 (docking score = − 270.15). (**R**) Docking result of CD44 and MDM4 (docking score = − 295.6). (**S**) Docking result of CD44 and LRP4 (docking score = − 231.56).

We used HDOCK to perform docking calculations to predict the preferred binding interactions between CD44 and the reported protein in the PPI network (Fig. 6A–S). The protein structure of VCAN (CSPG2) was not included in the database, so we did not simulate protein–protein docking between them. Table 2 showed the docking score and confidence score of the best docking model for CD44 and 19 proteins. The simulated docking results of CD44 and SGPP1(SPP1) showed the best stability (docking score = − 365.2). Secondly, the docking model formed by CD44 and SL9A1(SLC9A1) was stable (docking score = − 357.36).

**Table 2:** Molecular docking analysis of CD44 with the reported protein.

Gene	Protein	Docking score	Confidence score
SELE	LYAM2	− 243.65	0.8668
ROCK2	ROCK2	− 243.06	0.8654
SLC9A1	SL9A1	− 357.36	0.9844
HYAL2	HYAL2	− 316.46	0.9654
IGFBP3	IBP3	− 306.46	0.9581
ABCB5	ABCB5	− 335.79	0.9762
SPP1	SGPP1	− 365.2	0.9867
ABCC5	MRP5	− 327.86	0.9723
CD9	CD9	− 339.59	0.9779
TIAM2	TIAM2	− 297.61	0.9504
PDPN	PDPN	− 337.93	0.9772
SLC3A2	4F2	− 273.52	0.922
ITGA4	ITA4	− 283.97	0.9358
DMP1	DMP1	− 247.6	0.8757
DPP8	DPP8	− 294.82	0.9477
ITGB7	ITB7	− 293.78	0.9466
MMP7	MMP7	− 270.15	0.9171
MDM4	MDM4	− 295.6	0.9484
LRP1	LRP1	− 231.56	0.8363

## Discussion

We report the original study of CD44 expression in human bladder cancer and its relationship to tumorigenesis. We used public databases correlated its expression with immune checkpoint genes, immune infiltration. We found that patients with bladder cancer with high CD44 expression had worse survival than those with low CD44 expression (*P* < 0.05). In all subtypes of BLCA, the expression of CD44 in basal squamous was significantly higher than that in normal subjects. Indicating that CD44 was related to the prognosis and higher risk in patients with basal squamous bladder tumor. Furthermore, CD44 expression is significantly associated with Immune checkpoint genes (PD-L1, CTLA4, CD47). In vitro cell culture experiments, we found that CD44 can regulate PD-L1 at the mRNA level. Immune infiltration analysis showed that CD44 expression levels in BLCA were significantly correlated with immune infiltration levels of different immune cells. And the analysis showed that infiltration of macrophages, M2 macrophages means a worse prognosis for patients. Immunohistochemical (IHC) staining results further confirmed that the expression of CD44 was positively associated with the infiltration of macrophages and M2 macrophages (*P* < 0.05).

Intravesical infusion of chemotherapy or immune drugs after transurethral resection of bladder tumor (TURBT) is a commonly used adjuvant therapy for NMIBC^34^ and has been proven to be an effective method to remove residual tumor cells and prevent recurrence after surgery^35^. Treatment strategies for MIBC include neoadjuvant therapy, radiation therapy, and radical cystectomy (CR) or partial cystectomy^36^. Although the above clinical interventions can partially alleviate tumor recurrence and progression, a large proportion of patients progress to high-grade or metastatic disease, receive cisplatin-based cytotoxic chemotherapy, and have a poor prognosis (5-year progression rate of 0.8–45%)^36,37^. But in recent years, advances in our understanding of bladder cancer biology, along with large-scale gene expression and sequencing efforts, are leading to more accessible targeted clinical therapies and effective immunotherapies^38^. Furthermore, the recent success of immunotherapy regimens and a renewed focus on the tumor microenvironment (TME) has expanded the potential of targeted therapies. These advances are changing the strategy bladder cancer patients are treated, and improvements in overall survival and quality of life are expected to continue.

Increasing evidence suggests that CD44 is extensively overexpressed in other cancer types including gallbladder, prostate, ovarian, oral squamous cell carcinoma, and gastric cancer, correlating with aggressive biological behavior and a poor prognosis^39^. Interestingly, CD44 has not been overlooked in the bioinformatics analysis of BLCA. We found that the expression was a statistical difference in BLCA and paraneoplastic tissues through the TIMER database. Although CD44 expression was lower in paracancerous tissues, which provided interesting directions for our subsequent studies. We further analyzed survival differences such as Fig. 1B. TIMER is a good website that helps to visually analyze the differential expression of genes in various tumor types. In Fig. 1C, we performed subgroup analysis for different clinical stages, molecular subtypes, and histological subtypes. We sought to explore the specific reasons for the differences in CD44 expression between tumor and non-tumor tissues and to find the subgroup with the greatest differences. We found that CD44 was low expressed in tumors and highly expressed in non-tumors. This is consistent with the overall results we found using the TIMER database.

Papillary urothelial carcinoma is principally low-grade urothelial carcinoma, and nonpapillary urothelial carcinoma is mostly high-grade urothelial carcinoma. Expression differences exist between low-grade and high-grade bladder cancer due to differences in causative genes. This may explain the differential expression of CD44 between papillary and non-papillary tumors.

The immune system is one of the most influential cancer cell-extrinsic regulators of cancer biology. Similar to its physiological function, the immune system undertakes multiple tasks in a tumor-bearing host, with different immune cells playing different and sometimes opposite roles^40^. Immune cells in the Tumor immune microenvironment (TME) play an important role in tumorigenesis. These tumor-associated immune cells are known to have anti-tumor or tumor-promoting effects^41^. Our analysis reveals the expression of CD44 in BLCA was positively correlated with the expression of markers of macrophage, M1 macrophage, M2 macrophage, and myeloid-derived suppressor cell (r > 0.4). Tumor-associated macrophage (TAM) can be transformed to promote tumor cell growth and metastasis. Related studies have shown that TNF-α derived from M2-TAM can promote Epithelial mesenchymal transformation (EMT) and cancer stemness^42^. Based on the importance of TAM in cancer progression, in our IHC validation analysis, high CD44 expression of tumor cells was associated with high levels of CD68 + macrophages. These findings suggest that CD44 may regulate the recruitment of TAM and induce M2 Macrophage polarization in BLCA. And CD44 may promote the progression of bladder cancer by affecting the immunosuppressed M2 macrophages.

Immune checkpoint therapy has revolutionized the field of oncology and is an established treatment option for cancer patients. Tumor cells evade immune surveillance and progress through different mechanisms, including activation of immune checkpoint channels that suppress antitumor immune responses. By disrupting co-inhibitory signaling pathways, Immune checkpoint inhibitors (ICIs) reinvigorate antitumor immune responses and promote immune-mediated elimination of tumor cells^43^. The Food And Drug Administration (FDA) first approved an immune checkpoint inhibitor for CTLA4 in 2011, followed by FDA approval of another mab for PD1 in 2014^44^.

Based on the importance of immune checkpoints, we investigated CD44 expression was positively correlated with PD-L1, CTLA4, CD47 through the TIMER database. A study identified the cell-surface adhesion receptor CD44 as a key positive regulator of PD-L1 expression in triple-negative breast cancer (TNBC) and non-small cell lung cancer (NSCLC)^45^. Avelumab, durvalumab (anti-PD-L1), and nivolumab (anti-PD-1) are approved alternatives for advanced or metastatic BLCA. Consistent with the previous study, in vitro cell experiments showed that CD44 positively regulated PD-L1 in BLCA. However, PD-L1 expression in BLCA may also be regulated by other regulators, it requires further experimental research. Therefore, CD44 is a potential target to suppress PD-L1 function and can provide guidance for the clinical application of ICIs in BLCA.

Accordingly, CTLA-4 has been identified as a negative regulator of T cell function and proliferation, Drugs targeting CTLA-4 react on T cells^46^. Our bioinformatics results showed that CD44 expression was positively correlated with CTLA-4 expression. CD44 participates in the immune escape of tumor cells may be achieved by affecting CTLA-4 on T cells. However, further experimental validation is required to elucidate the relationship between CD44 and CTLA-4 on T cells.

PPI network analysis revealed that CD44 may play a key role in cell adhesion, tumor growth, drug resistance, cell differentiation, EMT, T cell activation, and immune function. Feature enrichment analysis result of the intersection of BLCA-related genes and genes that interact with CD44 was similar. The results of enrichment analysis showed that CD44 likely controls the PI3K-Akt signaling pathway in BLCA. In addition, CD44 may participate biological activity of biological activity of microRNAs, ECM-receptor interaction, Th17 cell differentiation.

We performed docking calculations to predict preferential binding interactions between CD44 and the proteins reported in the PPI network. Docking simulation results of CD44 and SGPP1 (SPP1) show the best stability. SGPP1 is an endoplasmic reticulum enzyme that dephosphorylates sphingosine-1-phosphate and has been shown to mediate cancer progression^47^. In addition, CD44 and SL9A1 (SLC9A1) docking simulation results showed high stability. The sodium/hydrogen exchanger 1 (NHE1), encoded by the human SLC9A1 gene (solute carrier family 9A1), is the major H + efflux mechanism for maintaining the alkaline pH_i_ of cancer cells^48,49^. The research reported SLC9A1 emerges as a marker for tumorigenesis and prognosis in gliomas^50^. And the research demonstrated both NHE1 regulate cell cycle progression in breast cancer cells^51^. In conclusion, CD44 may interact with SPP1, and SLC9A1 to promote certain tumor progression.

Several strategies have been developed to target CD44 and its ligands, including HA. For example, neutralizing antibodies against CD44 are in development and various stages of clinical trials. Bivatuzumab, KM201, U36, RG7356, and VF18 are some of the antibodies against CD44 in some studies^52^. Our study may provide new ideas for drugs targeting CD44.

Currently, bioinformatic analysis is an important tool for analyzing expression data and screening for target genes in many diseases^53^. Bioinformatics analysis can help us understand CD44 and BLCA more comprehensively. Through public online database bioinformatic analysis, we identified some important biological processes and pathways that probably play an important role in BLCA. Based on the results of these online databases, we further confirmed that the expression of CD44 was positively correlated with the expression of CD68 + and CD163 + by immunohistochemistry (IHC) staining. And we used in vitro experiments to verify CD44 is a positive regulator of PD-L1 in BLCA. In fact, this paper is just the beginning of a deeper study on the relationship between macrophages and CD44 that we are conducting. The study provides insights for further work that may require CD44 knockout and animal studies.

## Conclusions

Our results suggest that CD44 expression is associated with Immune checkpoint genes. And our results showed that macropahges, and M2 macrophages infiltration meant a worse prognosis. Samples with high CD44 expression had more macrophages, and M2 macrophages infiltration. And we tested this phenomenon in a cohort through BLCA tumor specimens. This was not previously reported in the literature. CD44 may be a key regulator of tumor macrophage infiltration and may be involved in M2 macrophage polarization. In addition, in vitro experiments confirmed that CD44 is a positive regulator of PD-L1 in BLCA.

We first proposed that macrophage infiltration may be a prognostic factor for BLCA. The expression of CD44 may be related to the immune escape of tumor cells because it has a regulatory effect on immune checkpoint genes. Our study provides a new idea for the prognosis and immunotherapy of BLCA patients by targeting macrophage infiltration and immune checkpoints.

Limitations of this study include that we did not validate some of the results of the bioinformatics analysis. These results require further in vitro and animal studies to confirm.

## Supplementary Information

Supplementary Figure S1.

## Data Availability

The raw data supporting the conclusions of this article will be made available by the authors, without undue reservation. The datasets for this study were obtained from public databases, including The Cancer Genome Atlas (TCGA) (https://cancergenome.nih.gov), UALCAN database (https://ualcan.path.uab.edu), Tumor Immune Estimation Resource (TIMER) database (https://cistrome.org/TIMER/), The GeneMANIA database (https://genemania.org), Metascape (https://metascape.org).
