# Supplementary material for: CD44 is a potential immunotherapeutic target and affects macrophage infiltration leading to poor prognosis
Source: Sci Rep. 2023 Jun 14;13:9657. doi: 10.1038/s41598-023-33915-4 (PMC10267145; doi:10.1038/s41598-023-33915-4)

Fig S1 (A) Tissue RNA expression of CTLA-4 in the Human Protein Atlas we bsite. (B) Tissue tissue specific cell types of CTLA-4 in the Human Protein At las website.

A

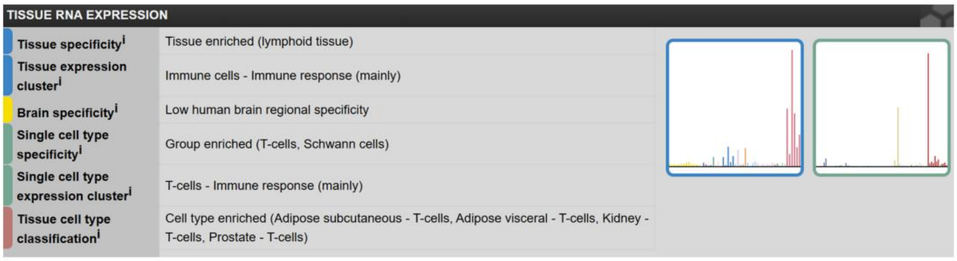

B

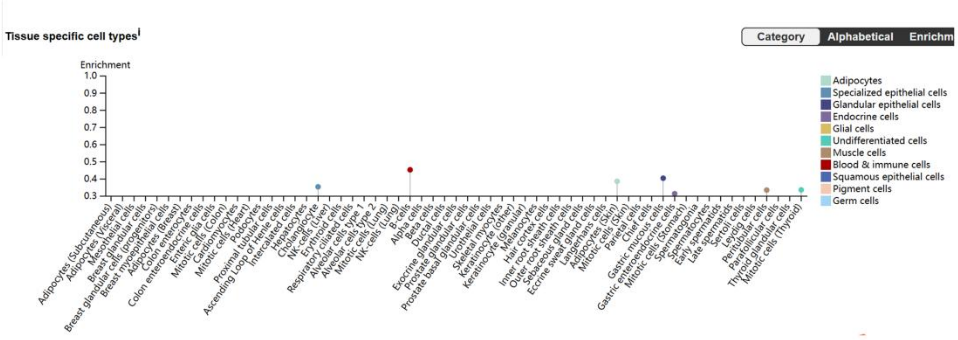

Supplement: Supplementary file 1 — Supplementary Figure S1. [file 41598_2023_33915_MOESM1_ESM.pdf]
